# Intersection of stem cell biology and engineering towards next generation *in vitro* models of human fibrosis

**DOI:** 10.3389/fbioe.2022.1005051

**Published:** 2022-10-20

**Authors:** Erika Yan Wang, Yimu Zhao, Sargol Okhovatian, Jacob B. Smith, Milica Radisic

**Affiliations:** ^1^ David H. Koch Institute for Integrative Cancer Research, Massachusetts Institute of Technology, Cambridge, MA, United States; ^2^ Institute of Biomedical Engineering, University of Toronto, Toronto, ON, Canada; ^3^ Toronto General Hospital Research Institute, University Health Network, Toronto, ON, Canada; ^4^ Department of Chemical Engineering and Applied Chemistry, University of Toronto, Toronto, ON, Canada

**Keywords:** tissue engineeering, stem cell, fibrosis, disease modeling, organ on a chip (OCC), biosensor, machine learning (ML)

## Abstract

Human fibrotic diseases constitute a major health problem worldwide. Fibrosis involves significant etiological heterogeneity and encompasses a wide spectrum of diseases affecting various organs. To date, many fibrosis targeted therapeutic agents failed due to inadequate efficacy and poor prognosis. In order to dissect disease mechanisms and develop therapeutic solutions for fibrosis patients, *in vitro* disease models have gone a long way in terms of platform development. The introduction of engineered organ-on-a-chip platforms has brought a revolutionary dimension to the current fibrosis studies and discovery of anti-fibrotic therapeutics. Advances in human induced pluripotent stem cells and tissue engineering technologies are enabling significant progress in this field. Some of the most recent breakthroughs and emerging challenges are discussed, with an emphasis on engineering strategies for platform design, development, and application of machine learning on these models for anti-fibrotic drug discovery. In this review, we discuss engineered designs to model fibrosis and how biosensor and machine learning technologies combine to facilitate mechanistic studies of fibrosis and pre-clinical drug testing.

## Background: Current challenges in modeling human fibrotic diseases for drug development

Human fibrotic diseases constitute a major health problem worldwide owing to their high incidence rate in most chronic inflammatory diseases (Wynn and Ramalingam, 2012). The data from the government of the United States suggests that fibrosis disorders are collectively responsible for 45% of deaths in the developed world (Wynn, 2004). Human fibrosis involves significant etiological heterogeneity and encompasses a wide spectrum of diseases affecting various organs. Typical fibrotic disorders include cardiac fibrosis (CF), idiopathic pulmonary fibrosis (IPF), primary myelofibrosis, scleroderma, as well as systemic fibrotic diseases including systemic sclerosis (SSc) and nephrogenic systemic fibrosis (Wells, 2013).

Fibrosis is generally initiated by a tissue repair process, followed by a scarring phase over the regeneration limit (Krenning et al., 2010). The replacement of normal parenchymal components by fibroblasts is often accompanied by a persistent release of fibrogenic cytokines, which in turn stimulates myofibroblast activation and deposition of extracellular matrix (ECM). Compromisation of organ function is a common paradigm in most fibrotic diseases following altered tissue micro-architecture. For example, many clinical cases of heart failure reflect histologic evidence of interstitial cardiac fibrosis (Fan et al., 2012). Cardiac fibrosis is associated with a progressive decline in cardiac performance, including diastolic dysfunction, reduced afterload, and arrhythmia (Leask, 2015). Similarly, idiopathic pulmonary fibrosis (IPF) remains a lethal condition, as it can lead to irreversible stiffening of lung tissue, distortion of the organ architecture, as well as decreased lung volumes and compliance (Lee et al., 2015).

Organ transplantation is the only therapeutic option for many end-stage diseases given the inefficiency and poor prognosis capabilities of fibrosis-modifying therapeutics (Rosenbloom et al., 2013). Drug discovery and development is a lengthy, costly, and risky process, reflected by substantial number of failed clinical trials and market withdrawals. The development of new pharmaceutical solutions for fibrosis has been extremely challenging as a comprehensive understanding of fibrosis etiology and evaluation of effective therapies have been hampered by lack of *in vitro* models that can faithfully recapitulate human pathophysiology.

Conventional disease models mainly rely on animal experiments and *in vitro* monolayer-based assays. Animal models, despite being the gold standards in current drug testing, present considerable discrepancies in both physiology and pathology compared to humans. Moreover, monolayer cell cultures are cost-effective alternatives for pre-clinical studies and have been widely used as established systems for pre-clinical drug testing. However, many of these models poorly replicate pathological factors responsible for disease onset and progression, because they lack of the structural and compositional complexity of native human tissues.

An ideal model for fibrosis studies and drug development should (Wynn and Ramalingam, 2012) produce a precise manifestation of disease phenotypes; and (Wynn, 2004) provide facile and reliable readouts that are important for fibrotic modeling. The major hurdle in new drug development is that the effects of some drugs on preventing or ameliorating the progression of fibrosis in practice cannot be reliably replicated in animals or 2D lab models. Therefore, it is hard to predict which compounds would have the optimal anti-fibrotic outcomes. A reliable testing system is one that approximates key features of the *in vivo* environment such as functional and structural properties, and molecular and metabolic signatures over the course of drug treatment. Ideally, it should also allow real-time and non-invasive tracking of multiplexed functional outputs (e.g., contractility and electrophysiological properties) in response to drug candidates under investigation.

**FIGURE 1:**
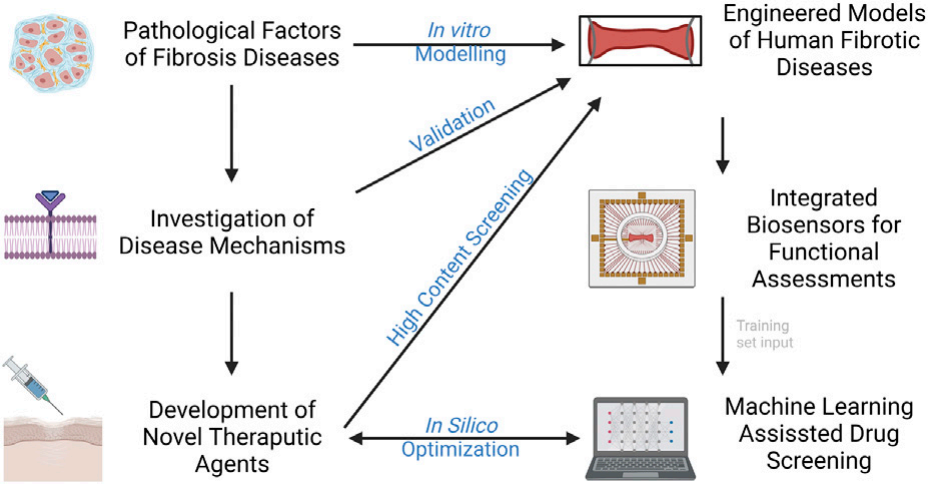
Current advances in modelling fibrotic diseases and therapeutic development. Engineered models of human fibrotic disease, incorporation of biosensors and machine learning would facilitate gaining insight into disease mechanisms and development of novel therapeutic agents in higher throughput.

Bioengineers are leveraging techniques in tissue engineering and microfabrication into the pipeline of human disease modeling. This will give rise to the next generation of *in vitro* models that are not only capable of modelling of fibrotic disease, but also test feasible therapeutic strategies. Organ-on-a-chip (OOC) technology, as a multi-disciplinary field that combines bioengineering, regenerative medicine, and platform manufacturing, has led to the establishment of authentic bio-systems to emulate human biology and diseases. This field has grown appreciably over the past decades with versatile approaches to generate disease- and patient-specific models for preclinical drug testing. Some of the most recent advances in modeling of fibrotic disease will be discussed, with an emphasis on the development of engineering techniques in the aspects of platform design, tissue construction, biosensor integration, and machine learning aided testing (Figure 1).

## Engineering of fibrotic models targeting distinct pathological factors

In different organ disorders, fibrosis arises from a variety of origins and involves multifaceted etiology. Replacement of fibroblasts, activation of immune cells, stimulation of growth factors, mechanical stress, and genetic background are some of the key factors that have been implicated in underlying fibrosis. Fibrosis occurs ubiquitously in the human body as a repair response to tissue injury. In healthy organs, the injury is followed by inflammation responses to cause immune cell infiltration and activation at the injury sites, including T cells, macrophages and neutrophils. These immune cells release profibrotic mediators to activate profibrotic signaling pathways such as TGF-β, WNT, hedgehog and PDGF, and initiate the transformation of vascular cells, resident fibroblasts, circulating fibrocytes and mesenchymal cells into myofibroblast-like phenotypes. Myofibroblast activation contributes to wound contraction and ECM protein deposition. The myoFBs often go through apoptosis after tissue repair in the context of physiological wound healing process. When persisted injury is in place, such process persists with elevated release of inflammatory cytokines and growth factors, abnormal myofibroblast activation, and excessive ECM deposition. The altered microenvironment including profibrotic mediators, ECM composition with increased stiffness and reduced compliance, in turn creates a positive feedback loop to aggravate the activation of myofibroblasts and prolong fibrosis (Distler et al., 2019). Genetic predisposition may also significantly contribute to the pathological activation of myofibroblasts and development of fibrosis.

To address the inherent complexities of fibrosis, novel engineering strategies have to recapitulate the intrinsic niche for fibrosis onset and progression. A rational experimental design to specifically probe individual or inter-related pathological facets and modulate their activities *in vitro* is essential for providing a better understanding of fibrosis pathogenesis and offering a more reliable testing microenvironment. Here we discuss the integration of the aforementioned factors into the model design to assist the precise manifestation of disease phenotypes.

### Cellular cues

Fibroblasts, as the cell population that constitutes majority of the stroma of tissues, constantly secrete extracellular matrix molecules and secrete factors to remodel the adjacent ECMs (Cox and Erler, 2011). More importantly, fibroblasts mediate the response to the inflammatory factors and matrix degradation products secreted by macrophages and endothelial cells (Wells, 2013). Fibroblast to myofibroblast activation is the key step in all forms of fibrosis, thus fibroblasts were widely maneuvered as the main switch to turn on fibrosis as well as the primary target for antifibrotic therapy. Scar-in-a-jar is a simplified tool for antifibrotic agent screening targeting fibroblast activation and ECM synthesis (Chen et al., 2009). However, this model is composed only of fibroblasts and does not reflect the physiological cellular diversity of human body. Co-culture of parenchymal cells and fibroblasts in engineered 3D platforms provides highly functional tissue constructs for use in investigational and therapeutic applications. For example, the model design strategy of the Biowire fibrotic tissues (Wang et al., 2019; Mastikhina et al., 2020; Wang et al., 2022) aims to reproduce the microenvironment involved in common forms of cardiac fibrosis, where the elevated presence of cardiac fibroblasts takes place after the loss of cardiomyocytes. Overpopulation of resident fibroblasts cause enhanced collagen content and tissue stiffening, which in turn activates progressive myofibroblast activation and aggravates more collagen deposition. In this fashion, fibrosis is mediated by a self-reinforcing positive feedback loop (Wang et al., 2019).

Endothelial cells play an integral role in tissue repair and contribute significantly to the development of fibrosis through cytokine secretion and fibroblast activation. Injured vasculature triggers endothelial cells to secrete profibrotic mediators such as TGF-β, connective tissue growth factor/CCN family member 2 (CTGF/CCN2), and plasminogen activator inhibitor-1 (PAI-1), which directly recruit and activate fibroblasts to produce ECM proteins (Leach et al., 2013). In addition, endothelial-to-mesenchymal transition (EndoMT) may occur during chronic wound healing, which further enriches the myofibroblast population and contributes to tissue fibrosis (Hashimoto et al., 2010).

**FIGURE 2:**
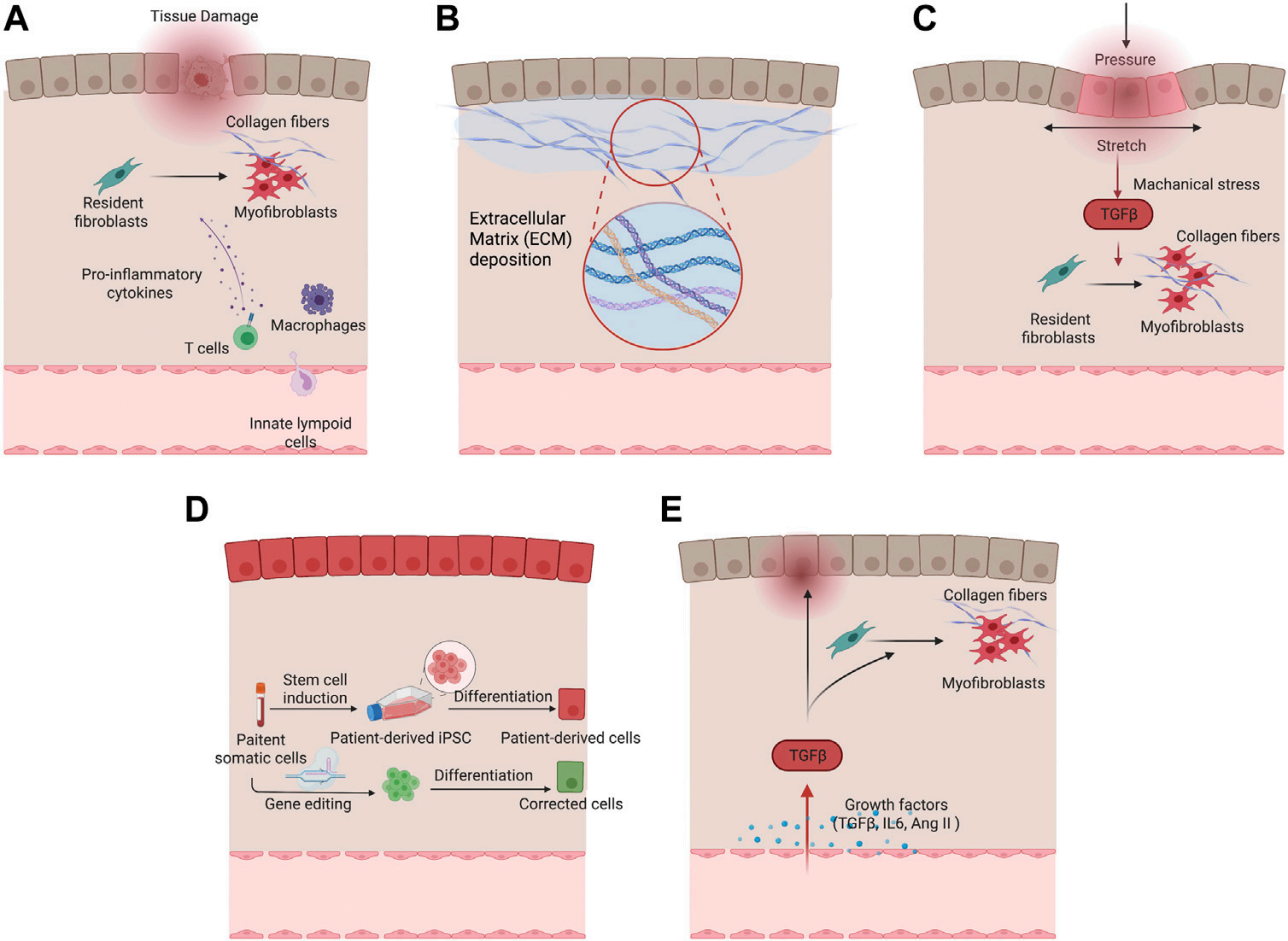
Different pathological factors of fibrotic remodeling. **(A)** Cellular factors underlying fibrotic remodeling. **(B)** Increased extracellular matrix deposition in fibrotic condition. **(C)** Mechanical stimulation drives myofibroblast activation and fibrotic remodeling. **(D)** Genetic predisposition in tissue and vascular cells with patient-derived cells serving for fibrotic disease modeling. **(E)** Growth factors including TGF-β and Ang II in fibrosis activation.

As a key process of wound healing, various immune cells engage in the manifestation of fibrosis after tissue injury. Macrophages are an important source of matrix metalloproteinases (MMPs) and inflammatory mediators that drive the initial tissue response after injury. Specifically, pro-inflammatory macrophages are predominant during the initial phase of wound healing, secretes pro-inflammatory cytokines, and produces MMPs for ECM degradation, which have potential antifibrotic effects. In later phases of wound healing, reparative macrophages secrete anti-inflammatory and profibrotic cytokines, such as Transforming Growth Factor β (TGF-β), Platelet Derived Growth Factor (PDGF), Insulin-Like Growth Factor 1 (IGF-1), and Vascular Endothelial Growth Factor α (VEGF-α), in response to inflammatory factors, such as interleukin 10 (IL-10) (Huang et al., 2020). With the production of these cytokines, reparative macrophages induce proliferation and recruitment of resident and circulating mesenchymal cells and activate these cells into myofibroblast phenotypes to produce ECM for wound contraction and tissue regeneration (Wynn and Vannella, 2016). The involvement of T cells has been shown in orchestrating fibrotic responses. T cell activation and the crosstalk between T cells and fibroblasts become increasingly important for recreating the niche and local environment for various fibrosis embodiments such as dermal fibrosis in burn patients (Zhang and Zhang, 2020). Incorporating endothelial cells and immune cells such as macrophages in engineered disease models can facilitate the precise manifestation of fibrotic phenotypes to study of the interplay of different pathological factors, and evaluation of systemic drug response. Figure 2A summarises cells involved in tissue fibrosis.

### Extracellular matrix cues

ECM cues refer to undergoing dynamic, structural and biomechanical changes in the extracellular matrix during fibrotic diseases. Because of the continuous activation of myofibroblasts, extracellular matrix proteins are excessively deposited in the injury sites. A hallmark of all fibrotic tissues is an increase in ECM stiffness. Major drivers of ECM stiffening include phenotypically converted myofibroblasts, activation of TGFβ pathway, and matrix cross-linking. For example, fibrotic myocardium is reported to have up to a 10-fold increase in Young’s modulus compared to normal myocardium (Jacot et al., 2010).

The increased stiffness is sensed by the resident cells through mechanotransduction by the surface integrin receptor and triggers downstream pathways such as Hippo followed by YAP/TAZ, which contributes to the upregulation of the profibrotic gene expressions (Kim et al., 2019). The profibrotic signaling pathways, such as PDGF, CTGF, and PAI-1, continue to promote the proliferation of myofibroblasts. Thus, targeting ECM mechanics, by preventing or reversing tissue stiffening or interrupting the cellular response, is an engineering approach to generate fibrotic disease models that offers potential targets for therapeutic intervention. Many studies aimed to incorporate and study this microenvironmental niche using organ-on-a-chip devices. Caliari et al. (2016) utilized a gradually softening hydrogel to investigate the hepatic stellate cell behavior during fibrosis regression and reinjure. Stellate cells were initially primed on tissue culture plastics to attain myofibroblast phenotypes and persisted when seeded onto the stiff hydrogel with Young’s modulus of 20 kPa. During the 2-week period of softening the hydrogel, reversal of myofibroblast phenotype was evident by reduced protein expression of alpha smooth muscle actin (α-SMA) and gene expression of YAP/TAZ signaling pathway. The re-stiffening of the hydrogel triggered reappearance of myofibroblast phenotype which supports the dynamic nature of this process and confirms the ECM as one of the critical microenvironment niches for *in vitro* fibrosis model.

Decellularized ECM may represent an ideal substrate for fibrosis modelling as it preserves the intrinsic biochemical and topographical micro-environments. ECM derived from hypertrophic myocardium in swine model was decellularized and reseeded with human cardiomyocytes (Sewanan et al., 2019). The resulting tissues demonstrated impaired twitch in comparison with the same cardiomyocytes seeded on ECM from healthy animals (Sewanan et al., 2019). Tissues grown on the diseased matrix exhibited prolonged contractions and poor relaxation. It has been suggested that both the mechanical properties and molecular compositions of ECM contribute to the impaired cardiac function. More recently, Shin et al. (2021) developed hybrid hydrogel bioinks containing partially digested porcine cardiac decellularized ECM and demonstrated the application in printing both healthy and fibrotic cardiac tissues with high shape fidelity and cell viability (Figure 2B).

### Mechanical cues

Organs that experience continuous mechanical stimuli, such as the heart and lung, can develop fibrotic phenotypes due to altered mechanical activity (Zhang et al., 2015; Yuan et al., 2018). Myofibroblast activation responsive to extensive strains was evident both *in vitro* and *in vivo* (Yuan et al., 2018; Kong et al., 2019). The activation of YAP/TAZ pathway and MAPK signaling pathways are responsible for the elevated α-SMA expression and myofibroblast transformation (Kim et al., 2019). Interestingly, among the three MAPK kinases (ERK, JNK, P38), only P38 can be activated through tensile stretching, which encourages myofibroblast activation (Zhang et al., 2003). When activated, myofibroblasts express α-SMA, which is incorporated into stress fibers through myosin light-chain phosphorylation *via* RhoA/ROCK pathway (Rouillard and Holmes, 2012). Moreover, mechanical stretching causes release latent TGFβ by opening the ECM bonded TGFβ1 straitjacket activate myofibroblasts to contract and change their mechanical microenvironment, resulting in a positive feedback loop (Rouillard and Holmes, 2012; Rosenbloom et al., 2013; Santos and Lagares, 2018; Roth Gregory et al., 2020).

Many engineered models incorporated external mechanical stimuli to facilitate pathological remodeling. In a three-dimensional microtissues model composed of fibroblasts in macroscopically engineered clefts, Kollmannsberger et al. (2018) discovered that tensile forces drive a reversible fibroblast-to-myofibroblast transition at the highly tensed growth front as the microtissue progressively closed the cleft, in analogy to closing a wound site. In another model, cyclic compressions with gradient magnitudes and tunable frequency were subjected onto gelatin methacryloyl (GelMA) hydrogels laden with CFs in a microdevice. Mechanical compression was observed to induce CFs proliferation and fibrotic phenotype transition, depending on the strain of mechanical load and myofibroblast maturity of CFs encapsulated in GelMA hydrogels (Yuan et al., 2018) (Figure 2C).

### Patient-specific genetic background

Patients with genetic diseases ranging from cystic fibrosis to systemic sclerosis, have genetic predispositions that put them at higher risk of tissue fibrosis. Although the state of art genetic sequencing technology allows high throughput screening and identification of genetic variants that contribute to fibrosis, these genetic variants have extremely high diversity among individuals (Lek et al., 2016). Therefore, a patient-specific *in vitro* model would provide a direct method to validate the correlation between genetic variances and functional deteriorations of fibrosis in patients, as well as to identify the specific disease mechanisms and personalized therapeutic targets (Figure 2D).

Patient-specific disease modeling is often performed by comparative assessments of patient-derived lines with their family-matched controls or gene-edited isogenic cell lines. The development of genome editing technologies has extended our ability to directly modifying genomic sequences. Recent advances in CRISPR-Cas9 system have enabled rapid genome editing in almost all eukaryotic cells with high efficiency and specificity. Up to now, CRISPR-Cas9 has been used to correct a wide spectrum of disorders, ranging from a defect associated with cystic fibrosis in human adult stem cells (Schwank et al., 2013) to a mutation in the calmodulin 2 (CALM2) gene with long QT associated syndrome in iPSC-cardiomyocytes (Limpitikul et al., 2017), leading to a remarkable functional rescue of disease triggered phenotypes.

In a lung-on-a-chip model, healthy and idiopathic fibrosis patient-derived lung fibroblasts were co-cultured with HUVECs in a microfluidic-based platform (Mejías et al., 2020). Strikingly, the idiopathic fibrosis tissues have a high baseline of α-SMA and do not react to TGF-β or clinically used antifibrotic drug (Pirfenidone), whereas the healthy tissues showed superior responses. In a pancreas-on-a-chip model, patient-derived ductal epithelial cells and islets were co-cultured in two-cell culture chambers separated by a thin layer of porous membrane. When compared to the healthy control, cystic fibrosis related insulin disorder was captured in the disease model (Shik Mun et al., 2019). Despite the lack of in-depth investigation of disease mechanisms, these proof-of-concept studies demonstrated the potential of these genetically edited models in the application of precision medicine and drug discovery.

### Growth factors

Many studies, both *in vivo* and *in vitro*, concluded that TGF-β is the predominant pathogenic factor that drives fibrosis. Upon tissue injury, TGF-β is released from latent TGF-β binding protein (LTBP) complex in ECMs. Macrophages can also produce TGF-β during the inflammation process. After binding with receptors on the cell surfaces, Smad 2, 3, and 4 will form complex and translocate to the nucleus to upregulate transcription of profibrotic molecules, such as α-SMA, collagen I, and tissue inhibitor of matrix metalloproteinases (TIMP) hence induce myofibroblast transformation of the cells (Meng et al., 2016). Thus, as a direct modulator, TGF-β has been used in various *in vitro* models to initiate myofibroblast transformation. The common methodology is to subject the culture to TGF-β treatment to stimulate the transformation of resident fibroblasts into myofibroblasts and evaluate the functional and structural changes *in vitro* (Oakley et al., 2019).

Lee et al. (2019a) established self-aggregated cardiac microtissues by co-culturing hESC-MSC and hESC-CM in order to transform of MSCs toward cardiac fibroblast phenotype. After 2 weeks of TGF-β treatment, cardiac microtissues showed impaired contractile behavior, mitochondria deformation, and smaller tissue sizes with a significant portion of CMs going through apoptosis. They also observed increased myofibroblast activation and collagen deposition in the TGF-β treated group, which are consistent with global transcription analysis. Comparable results were presented in the study by Sadeghi et al. (2017), where fibrosis remodeling was activated by the exogenous addition of TGF‐β1 in a simplified three‐dimensional (3D) hydrogel platform. In a more sophisticated *in vitro* platform developed by Mastikhina et al. (2020) papillary-like cardiac tissue was formed under uniaxial tension, where the force of contraction and tissue stiffness changes were presented to further validate fibrotic nature of the tissues. TGF- β in this system was also shown to significantly reduce the force of contraction and increase local and global tissue stiffness in addition to the genetic and protein level alteration. In contrast to results in 2D culture system, Pirfenidone was shown to reduce the profibrotic gene expressions and tissue stiffness. In a lung-on-a-chip model, Pirfenidone was also shown to minimize the TGF- β induced fibrosis, in terms of α-SMA expression, and passive tensions (Asmani et al., 2018). These results demonstrated the superiority of a more complicated *in vitro* system with precise recapitulation of clinical outcomes.

Angiotensin II (Ang II), the main peptide of Renin-Angiotensin System (RAS), is believed to induce progressive deterioration of organ function. Its upregulation is implicated in various pathological conditions such as cardiac hypertrophy and fibrosis, offering another important mediator for the disease model development. Ang II can induce fibrosis indirectly by activating proinflammatory mediators such as cytokines, chemokines, adhesion molecules, and directly by regulating extracellular matrix synthesis and degradation, increasing tissue stiffness and leading to the impaired function. Horton et al. (2016) utilized a cardiac platform composed of neonatal rat cardiomyocytes on muscular thin films (MTF) to study tissue dysfunction in response to Ang II exposure. This model mimics many features of disease observed *in vivo*, with pathological gene expression profiles including over-expression of B-type natriuretic peptide (BNP), Rho GTPase 1, and T-type calcium channels, as well as increased proarrhythmic early after depolarization events, along with significantly reduced peak systolic stresses. In a recent study from our group, a Biowire II disease model was constructed to recapitulate myocardial response to Ang II in a temporal manner and evaluate compounds that target Ang II-induced cardiac remodeling (Wang et al., 2021a).

Many other pro-fibrotic mediators recently came into focus, such as IL-10 cytokine family members. These pro-inflammatory cytokines exert host defense mechanisms and facilitate tissue healing (Jennings et al., 2015). Recent studies reveal a close association between IL-10 family cytokines and fibrotic diseases including Interstitial lung disease (Sziksz et al., 2015), renal fibrosis (Jennings et al., 2015), and cardiac fibrosis (Zimmermann et al., 2012). Pro-fibrotic hypoxia signaling pathway was also shown to correlate extensively with fibrosis (Senavirathna et al., 2018; Aquino-Gálvez et al., 2019; Kuo et al., 2019). It has been shown to significantly upregulate the α-SMA expression in idiopathic lung fibrosis patient-derived cells and healthy controls through HIF-1α & HIF-2α (Figure 2E).

## Engineered models for studying human fibrotic diseases

Engineered platforms for *in vitro* fibrosis disease modeling have been created to recapitulate chemical, mechanical, and biological conditions in fibrosis of various organs (Tissue-engineered disease models, 2018). Human-induced pluripotent stem cell (hiPSC) technology presents an unprecedented opportunity for disease modeling in association with these platforms (Brandão et al., 2017; Zhang et al., 2018a; Zhao et al., 2019a). Human-based fibrosis-on-a-chip models have been designed to mimic the pathological niche *in vivo* (Besser et al., 2018; Tissue-engineered disease models, 2018; Ulmer and Eschenhagen, 2020). These models have shown promise for deciphering fibrosis mechanisms and discovering effective drugs. Moreover, the advances in differentiation of human pluripotent stem cell hiPSCs into three the germ layer lineages facilitate the development of three-dimensional hiPSC-derived organoid models that recapitulate the native organ with remarkable fidelity (Devarasetty et al., 2018). Organoids, which are self-organizing tissues, provide a new powerful tool for disease modeling and have been increasingly adopted since 2009 (Wörsdörfer et al., 2020). When cultured within an extracellular matrix and provided with suitable culture media and growth factors, they can self-organize into 3D structures that resemble miniature organs composed of various cell types. Organoid models offer a unique approach for studying pathological conditions including fibrosis. Most recent *in vitro* OOC and organoid disease models in the context of various fibrotic disorders in the past 5 years are discussed below.

### Cardiac fibrosis

Our group developed a heart-on-chip model of human myocardial fibrosis and used it to establish a compound screening framework. In this platform termed Biowire II, cardiac tissues were cultured around a pair of poly [octamethylene maleate (anhydride) citrate] (POMaC) wires to recapitulate contractile, biomechanical, and electrophysiological complexities of fibrotic myocardium (Table 1). The structural and mechanical properties of the fibrotic tissues were tailored by tuning the cellular composition. Hallmarks of fibrotic myocardium characterized such as myofibroblast activation, excessive collagen deposition, tissue stiffening, impaired contractile and electrical properties, and concomitant arrhythmogenesis were obtained. Additionally, we constructed a heteropolar integrated model where fibrotic and healthy cardiac tissues were coupled together to capture the regional heterogeneity of scar lesion, border zone, and adjacent healthy myocardium (Wang et al., 2019). Other groups have designed similar heart-on-chip models with tissues attached to polydimethylsiloxane (PDMS)-based wires and observed similar fibrotic responses such as increased tissue stiffness, BNP secretion and collagen deposition (Mastikhina et al., 2020).

**TABLE 1:** Recent models of studying fibrosis *in vitro*. Current models have been able to recapitulate various aspects of fibrosis in cardiac, liver, skeletal muscle, lung and intestine engineered tissues. All figures are reproduced under the terms of CC-BY license.

Organ	00AC model	Key advances	Main readouts	Drug screening	Stage of translation	Opportunity for future works	References
Heart 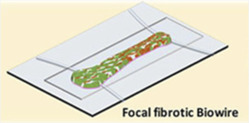	Biowire II	Heteropolar integrated model of healthy and fibrotic tissue	Active and passive force. Excitation threshold (ET). Maximum capture rate (MCR). electrical propagation, tissue compaction force frequency relationship (FFR). Collagen deposition	Furin inhibitors (PCI, Fil, and Fill)	Proof-of- concept	Should Include all myofibroblast origins for phenotypical heterogeneouty	Wang et al. (2019) ACS central science
		Non-invasive readouts of tissue Constraction				Incorporating automated cell seeding or 3D printing technologies to prevent patterning Inconsistencies	
Lung 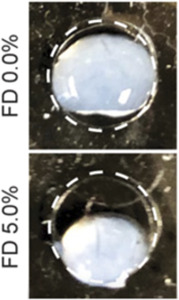	3D model with multi-component hydrogel	3D fibrous structure of interstitial tissue in idopathic pulmonary fibrosis (IPF)	Collagen deposition, tissue compaction, cellular remodelling	YAP inhibitor (dimethyl fumarate), IPF therapeutics (nintedanib. pirfenidone and their combination)	Proof-of-concept	Matrix remodelling over time	Matera et al. (2020) Science Advances
						Crosstalk among relevant cell types i.e., epithelial cells macrophages and endothelial cells	
Liver DAPI COL1A1 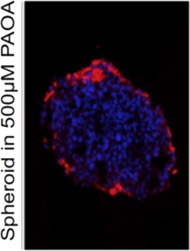	Spheroid	Enables studying molecular mechanisms underlying lipid accumulation during liver fibrosis	Matrix topography	Potential therapeutics of NASH (Iiraglutide. elafibranor, vitamin E and obeticholic acid)	Proof-of-concept leading to clinical evaluations	Matrix stiffness at different stages of fibrosis	Pingitore et al. (2019) International Journal of Molecular Sciences
			myofibroblast activation			Crosstalk among relevant cell types i.e., epithelial cells, macrophages and endothelial cells	
Skeletal Muscle 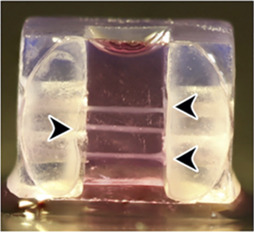	Vacularized sheet of human muscle-derived fibroblasts and muscle cells	Incorporation of endothelial cells within model	Collagen fiber deposition	N/A	Proof-of-concept	Functional assessments. i.e., changes of muscle contractility. Additional elements to study i.e., matrix compositions, and inflammatory cytokines	Bersini et al. (2018) Cell reports
		Enables studying the mechanism of muscle-specific fibroblast recruitement					
		Physiologically relevant architecture					
Intestine 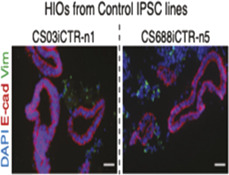	Organoids	Personalized approach for intestinal fibrotic response using hiPSCs	Flow eytometric assessment of cell populations	N/A	Proof-of-concept	Benchmarking to the clinical data-	Estrada et al. (2021) Inflammatory Bowel Diseases
			Assessment of N-cadherin, COL5A1. COL1A1 and TIMP1 secretion after TGF-β treatment			Further evaluation of fibrosis hallmarks. i.e., functional and ECM changes	

Another engineered *in vitro* model of human cardiac fibrosis was developed using human fetal cardiac fibroblasts (hfCF) encapsulated in a gelatin methacryloyl (GelMA) hydrogel. Subsequent TFG-β1 stimulation in hfCF-laden GelMA resulted in a fibrotic phenotype characterized by myofibroblast activation and ECM accumulation. This fibrotic model was utilized to investigate the effect of cardiac progenitor cells (CPC) on the disease process, supporting previous *in vivo* findings of the anti-fibrotic potential of CPC in conjunction with hfCF (Bracco Gartner et al., 2019).

### Liver fibrosis

Davidson et al. (2017) developed a micropatterned tri-culture (MPTC) platform that displayed early fibrosis-like hepatic dysfunctions associated with non-alcoholic steatohepatitis (NASH). Clinically relevant hepatic dysfunctions caused by activation of human stellate cells (HSCs) were captured in their multi-well microfabricated platform. The disease model was used for investigating mechanisms in NASH/fibrosis and screening of therapeutics targeted towards the early stages of fibrosis.

In another study, an *in vitro* model of human liver fibrosis was developed in a hepatic organoid system. Multilineage 3D spheroids composed of hepatocytes (HepG2) and hepatic stellate cells (LX-2) were constructed and exposed to free fatty acids to induce steatosis and fibrosis. This fibrotic organoid model was used to study molecular mechanisms underlying lipid accumulation induced fibrogenesis and screen compounds that are in clinical trials for the treatment of NASH (Pingitore et al., 2019) (Table 1).

### Pulmonary fibrosis

In the area of pulmonary fibrosis, Matera et al. (2020) developed a 3D model composed of multi-component hydrogel that recapitulates the 3D fibrous structure of interstitial tissue regions in idiopathic pulmonary fibrosis (IPF). Key design criteria were determined by referring to mechanical properties of fibrotic interstitial tissue in a bleomycin-induced lung injury model in mouse. The microengineered pulmonary interstitial matrix resembles mechanical and structural features of fibrotic tissue, as well as key biological events underlying IPF progression. This model was then applied to study the correlation of altered matrix topography and myofibroblast activation, and to probe fibroblast-ECM interactions. Furthermore, multiple antifibrotic agents were tested in the engineered fibrotic pulmonary interstitial matrices and exhibited aligned antifibrotic efficacy with the previous clinical studies (Table 1).

Another model of idiopathic pulmonary fibrosis was constructed using 3D organoids. Gene-edited embryonic stem cell (ESC)-derived lung organoids were generated and showed to recapitulate important phenotypes of Hermansky-Pudlak syndrome-associated interstitial pneumonia (HPSIP). They further validated the hiPSC-derived lung organoids as a useful tool to identify potential drug targets. Genome-wide expression analysis from the HPSIP mutant fibrotic organoids deciphered the crucial role of interleukin 11 (IL-11) in the fibrotic process (Strikoudis et al., 2019).

### Fibrosis in other organs

Fibrosis has been successfully captured in a wide range of engineered *in vitro* models of other organs and tissues. Bersini et al. (2018) reported the generation of a mesoscale human muscle model. This model is composed of differentiated human muscle fibers enveloped by a sheath of human muscle-derived fibroblasts and supported by a vascular network with mural-like cells. This vascularized skeletal muscle model was used to capture key physiological features of fibrosis. Abnormal fibrotic deposition of ECM proteins and myofibroblast activation were observed when Duchenne muscular dystrophy (DMD) fibroblasts were embedded in the 3D vascularized muscle environment (Bersini et al., 2018) (Table 1). Estrada et al. (2021) reported the generation of human intestinal organoids derived from patient derived-induced pluripotent stem cells. These 3D organoids provided a personalized approach for modeling intestinal fibrotic responses *in vitro* (Table 1). Tissue-engineered skin is an emerging tool for identification of novel therapeutic interventions for treating skin injuries and hypertrophic scars, especially for burn patients (Vig et al., 2017). In a work by Varkey et al. (2013) layered fibroblasts and keratinocytes were co-cultured on collagen-glycosaminoglycan scaffolds to build an engineered skin model. This model was used to investigate the role of keratinocytes in fibrotic remodeling.

## The incorporation of built-in biosensors for functional assessment

There has been a growing interest in application of novel biosensors in engineered *in vitro* systems. In order to use the microphysiological models to expedite high content and high-throughput compound screening and assess toxicity or evaluate the effectiveness of new anti-fibrotic drugs, their readouts shall be facile and reliable. Biosensors are physical transducing systems that are integrated with the biological components of the engineered models. Implementation of such transducing system enables quantitative study of the interaction between biocomponent and tested therapeutic compounds. An ideal biosensing system should provide a means to collect the drug testing data in real-time non-invasively. To this end, different strategies are applied to incorporate biosensors into engineered OOC systems and performing *in situ*, real-time and automated analyses.

Great efforts have been undertaken to integrate various types of biosensors into OOC platforms. Based on their distinct analytical approaches, OOC biosensors can be classified into several types including electrochemical, optical, and piezoelectric biosensors, etc. (Fahliyani et al., 2020). In the context of fibrotic remodeling, progressive stiffening, contraction of tissue, and the decline in tissue compliance are the key readouts of different fibrosis models. We will mainly focus on primary readouts for anti-fibrotic drug study and summarize existing biosensing systems for functional assessment of tissue contraction, stiffness, and electrical properties in this section.

### Biosensor for measuring fibrotic tissue contraction

Tissue contraction is an important determinant of fibrotic phenotype. In fibrotic tissues, myofibroblasts, containing cytoplasmic bundles of microfilaments or stress fibers, are interconnected by gap junctions, and are also connected to the extracellular matrix by a specialized structure called fibronexus, thereby play a pivotal role in tissue contraction. During the fibrosis process, myofibroblasts are responsible for tissue remodeling both by tractional forces and contraction.

By utilizing elastic materials like silicone posts or elastic wires in fabrication of optical biosensors, contractility can be calculated based on deformation of the substrates. For example, the Biowire platform uses wire deflection to continuously measure force values and dynamics, capturing declined force amplitude and increased tractional force in a fibrotic disease model. Non-invasive functional readouts are realized on the basis of the deflection of the intrinsically fluorescent polymer (Wang et al., 2022). By assessing the dynamic change in passive tension, the authors evaluated antifibrotic efficacy of the small-molecule inhibitor of proprotein convertase furin and proofed potential application of the sensing system in preliminary compound screening (Wang et al., 2019; Wang et al., 2022).

**FIGURE 3:**
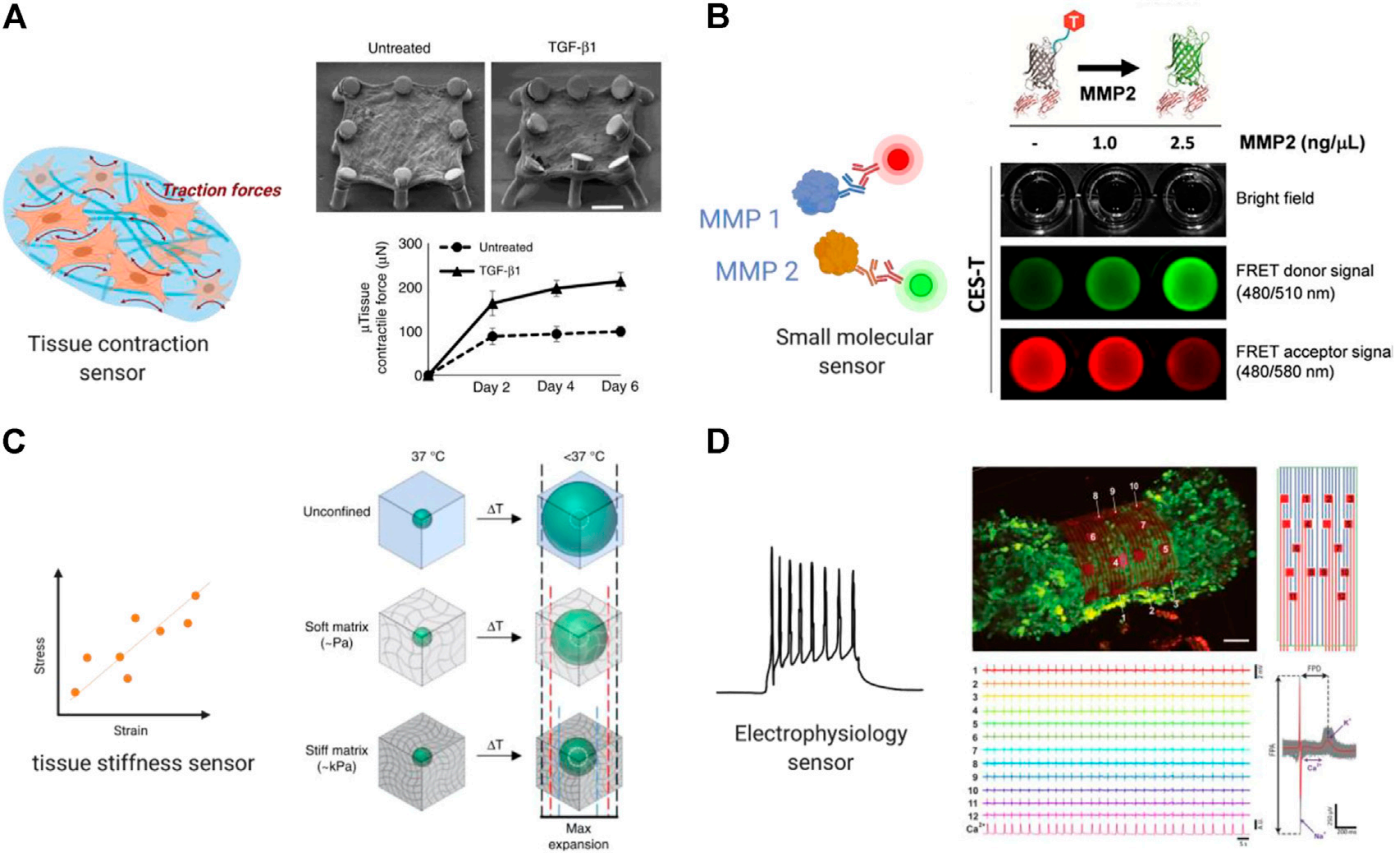
Built-in biosensors for anti-fibrotic drug screening. **(A)** Micropillar sensors integrated in a membranous human lung microtissue array to measure spontaneous contraction force generated by fibrotic tissues (Asmani et al., 2018). Reproduced under terms of the CC-BY license. **(B)** A microgel-based biosensor for the direct measurement of mechanical stiffness in local tissues (Mok et al., 2020). Reproduced under terms of the CC-BY license. **(C)** An extracellular FRET biosensor for the visualization of MMP2 activity in a 3D culture system (Lee et al., 2020). Reproduced under terms of the CC-BY license. **(D)** A self-rolled biosensor array to investigate electrogenic cell behavior in 3D human cardiac spheroids (Kalmykov et al., 2019).

In another study, Asmani and coworkers developed a membranous human lung microtissue array, where the spontaneous contraction force generated by individual microtissues was determined from the deflection of micropillars based on cantilever bending theory. They successfully captured progressive stiffening and contraction of alveolar tissue during lung fibrogenesis, and provide proof of principle for using this system for phenotypic analysis of the therapeutic efficacy of two PDA-approved anti-fibrosis drugs (Asmani et al., 2018) (Figure 3A).

### Biosensor for measuring tissue stiffness and mechanical properties

Tissue stiffness is a critical physical characteristic and plays a significant role in many biological responses including fibrosis (Wells, 2013). Due to excessive ECM deposition, tissue stiffening is one of the most direct readouts in all kinds of organ fibrosis. Therefore, extracting the drug effect on tissue stiffness is of particular interest for evaluating potential anti-fibrotic compounds.

Various *in situ* biosensors for monitoring cell- or tissue-level stiffness have been developed. Lam et al. (2012) developed a whole-cell cell stiffness sensing system with a subcellular spatial resolution, by using a cell stretching device that allowed quantitative control and real-time measurements of mechanical stimuli and cellular biomechanical responses. This platform is composed of a microfabricated array of silicone elastomeric micro-posts integrated onto a stretchable elastomeric membrane. Changes in micro-post top positions during mechanical stretches were recorded and converted to cell stiffness based on the data of local cell stretching forces and cell area increments.

Mok et al. (2020) developed a thermoresponsive microgel-based biosensor that can be dispersed or injected into tissues and optically tracked to measure mechanical stiffness in tissues. Based on the size-changing of the microgel, internal mechanical profiles of live multicellular spheroids were mapped at high resolutions to reveal spatial rigidity across the tissues. This biosensor also showed promising results in mapping tissue stiffness in an *in vivo* mouse model of breast cancer progression (Figure 3B).

In a more recent work led by Emon et al. (2021) a high-resolution sensor that allows self-assembly and culture of 3D tissue models was described. This sensor was applied to detect single and multiple cell forces in 3D matrices over long-term culture with a high resolution of 1 nN. The design also allowed quantification of the changes in stiffness of the tissue remodeled by the cells. This system originally aimed at testing tissue stiffness in tumor microenvironment but has great potential in fibrotic study and the development of anti-fibrotic compounds.

### Biosensor for molecules detection

It is important to enable real-time detection of functional protein molecules in the fibrotic process released from different cell populations and phenotypes to understand the fundamentals of cell responses for disease modeling and drug screening applications. Some of the key molecules that are commonly assessed are MMPs, and inflammatory cytokines (Hasan et al., 2014). MMPs are a group of enzymes in the proteinase family, in concert are closely related to the dynamic degradation of most extracellular matrix proteins during organogenesis, as well pathological remodeling process including fibrosis. Thus, MMPs can serve as real-time biomarkers under fibrotic state and in response to different treatments, which can be measured and quantified using a biosensing system.

Due to low concentration of many biomarkers in the microphysiological system at nanogram and picogram levels, it is important to develop highly sensitive biosensing systems and signal amplification approaches. To this end, Förster or fluorescence resonance energy transfer (FRET) based biosensors were developed to measure molecular events that occur relatively fast in single cells or engineered tissues with high signal to noise ratio and spatio-temporal resolution (Liu et al., 2020).

Utilizing FRET imaging, Ouyang et al. (2008) developed a biosensor that has its sensing element anchoring at the extracellular surface of cell membrane to visualize dynamic MT1-MMP activity in live cells with subcellular resolution. Constructed with a substrate sensitive to MT1-MMP, this biosensor was able to capture drastic FRET changes induced by epidermal growth factor (EGF) in migrating cancer cells, *via* a process dependent upon an intact cytoskeletal network. In a follow-up study, the authors further developed two membrane-anchored FRET biosensors of different spectra and used the system to concurrently visualize the activities of Src and MT1-MMP in a single live cell. The result delineated the temporal and spatial differences between MT1-MMP and Src activation upon EGF stimulation (Ouyang et al., 2010).

In a more recent work, a similar approach using cleavage-based FRET reporters was reported to detect metalloproteinase-2 (MMP2) activity in living cell culture. In this report, Lee et al. (2020) developed an extracellular FRET reporter which consists of an ECM collagen-binding protein and a FRET coupler of an enhanced green fluorescent protein (eGEF) and a small dye molecule. The extracellular FRET reporter can bind to the collagen matrices and enables the visualization of MMP2 activity through fluorescence changes. This system showed feasibility in detecting protease activity in a 3D culture system. (Figure 3C) Many challenges still exist in determination of disease markers using biosensors, such as nonspecific binding and the small size of the target which can be overcome in future studies.

### Biosensor for detecting electrical signals

Electrophysiology is an important feature of electroactive tissues including cardiac and neuronal tissues. The fibrotic process is particularly detrimental as the heart is characterized by limited regenerative and repair capacity. In the case of cardiac fibrosis, overpopulated cardiac fibroblasts result in ECM deposition and hence altered cardiomyocyte electrophysiology and disrupted electrical impulse conduction of the myocardium (Ramanathan et al., 2006). The activated myofibroblasts can be coupled to cardiomyocytes through gap junctions and result in partial depolarization of cardiomyocytes (Thompson et al., 2011). Therefore, integration of electrical biosensors can provide targets for potential drug therapies of cardiac fibrosis. An ideal cardiac OOC platform should provide multiplexed measurements of electrophysiological aspects. Concerted efforts in sensor integration in OOC platforms will open a new avenue for spatiotemporal exploration of cardiac disease.

Microelectrode array (MEA) is a widely used approach for recording electrical signal as it can be easily integrated in microphysiological systems. Traditional MEAs are confined to 2D substrates that render spatiotemporal electrical recordings of a single plane. More recently, 3D bioelectrical interfaces were reported, enabling a more complex electrophysiological readout. Kalmykov et al. (2019) developed a self-rolled biosensor array using either built-in active field-effective transistors or passive microelectrodes to interface with encapsulated 3D human cardiac spheroids to investigate electrogenic cell behavior. These arrays provided continuous and stable multiplexed recordings of field potentials, supported with simultaneous calcium imaging (Figure 3D).

Cardiac conduction is another important functional readout of the heart and is often impaired by fibrotic remodeling (Thompson et al., 2011). Optical mapping is the conventional method to measure electrical propagation and arrhythmias. However, the fluorescent dyes induce phototoxicity in longer term application, therefore optical mapping is typically performed as end-point analysis. MEA is also accompanied by low spatial resolution and low signal-to-noise ratio. The development of cytotoxicity-free methods for real-time *in situ* recording of conduction velocity is of increasing research interest. Dou et al. (2020) developed a label-free method based on defocused brightfield imaging to quantify cardiac volume (CV) by analyzing centroid displacements and contraction trajectories of each cardiomyocyte in a 2D iPSC- cardiomyocytes culture without effecting cell viability.

Beyond the cardiac system, integrated electrodes are also applied in other organ models. These applications include the assessment of barrier integrity of cultured cells using trans-endothelial electrical resistance (TEER) measurements. Farooqi et al. (2021) explored TEER measurement’s utility for monitoring liver fibrosis development in a dynamic cell culture microphysiological liver system. A glass chip-based embedded TEER and reactive oxygen species (ROS) sensors were employed to gauge the effect of TGF-β1 within the microphysiological system, which promotes a positive feedback response in fibrosis development.

Overall, chip embedded electrochemical sensors could be used as a promising substitute for conventional end-point assays for studying fibrosis in microphysiological systems. In future, this fast-developing field has great value in illuminating mechanisms of diverse fibrotic diseases and accelerating discovery of novel anti-fibrotic drugs.

## Potential Therapeutic Targets for Fibrosis

**FIGURE 4:**
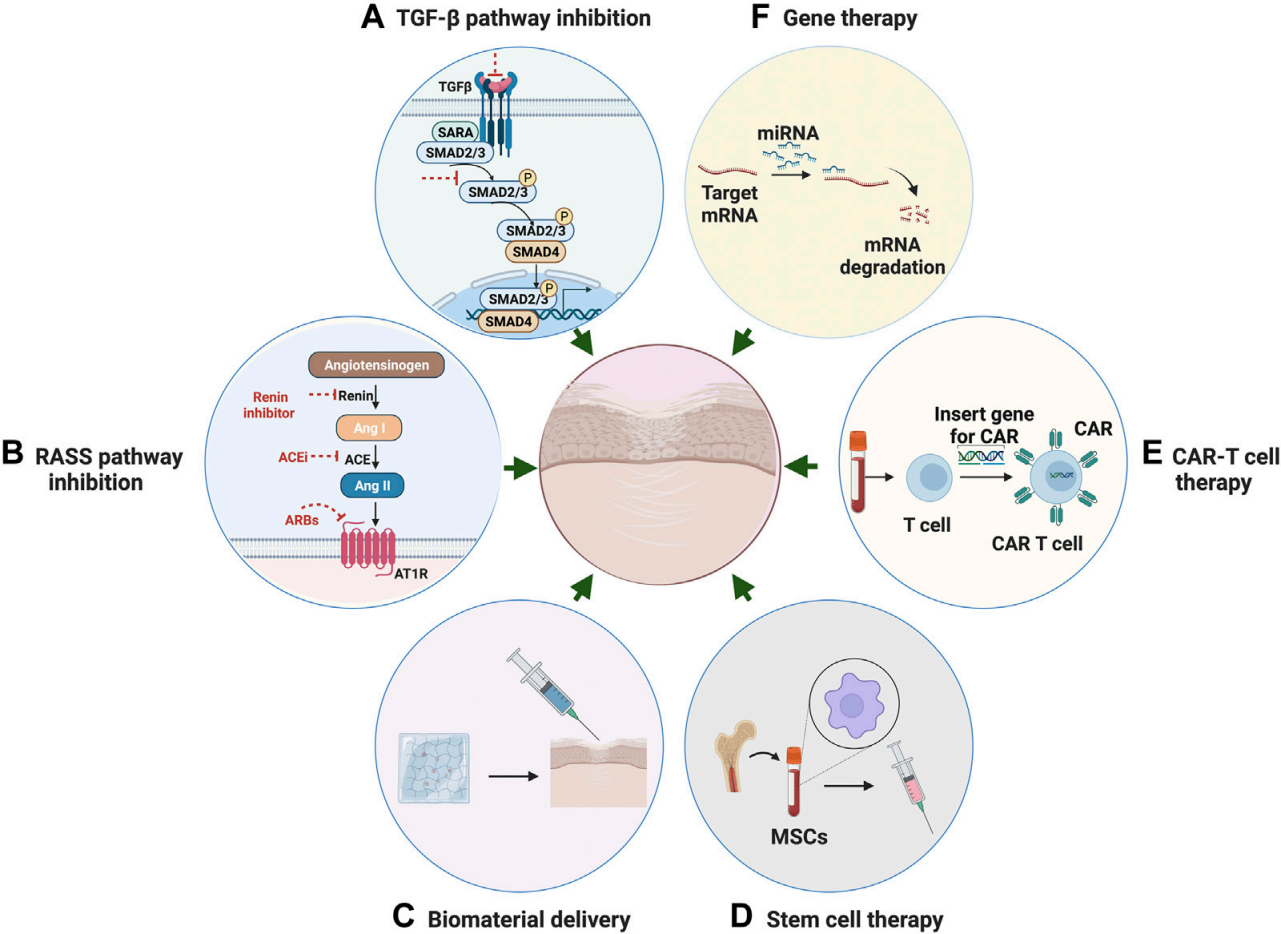
Potential Therapeutic strategies for fibrosis. **(A)** Anti-fibrotic therapeutic strategies through inhibition of TGF-β pathway. **(B)** Therapeutic strategies through inhibition of RAS system. **(C)** Targeted anti-fibrotic therapeutic approach through biomaterial-based delivery. **(D)** Stem cell therapy with mesenchymal stromal cells for the treatment of fibrotic disease. **(E)** CAR T-cell therapy using engineered T cells for the treatment of fibrotic disease. **(F)** Gene therapy through mediating miRNA as a novel approach for anti-fibrosis treatment.

Fibrosis represents a major health problem worldwide, yet there are still limitations in current knowledge of the disease mechanisms. Development of antifibrotic therapies relies on comprehensive understanding of profibrogenic mechanisms in organ-specific systems. Due to heterogeneity in disease etiology and phenotypes, and lack of clinically validated biomarkers, there is a no effective disease-modifying therapeutic agents specific to fibrosis. Conventional treatments rely on pharmaceutical compounds targeting inhibition of fibrosis-related molecular pathways. Various potential novel strategies have been investigated to combat fibrotic tissue damage in different organs (Figure 4).

### Transforming growth factor-related pathway inhibition

In the past few decades, research has highlighted potentially targetable pathways and related individual factors involved in the fibrogenesis process. These include transforming growth factor-beta (TGF-β), which has long been recognized as a master activator in fibrosis (Meng et al., 2016). TGF-β acts on multiple cell types to drive pathogenesis of fibrogenic responses in several different organs. TGF-β-induced fibrosis is activated through canonical Smad-dependent or non-canonical Smad-independent pathways and is modulated by various coreceptors and complex interacting networks (Meng et al., 2016).

There is a limited therapeutic feasibility and unpredictable off-target effects in direct targeting of TGF-β, due to the functional pleiotropy of this growth factor in a wide variety of complex biological processes. Recent discoveries in fibrotic pathways and factors regulated by TGF-β have facilitated the identification of alternative therapeutic targets and showed promise in achieving an attainable inhibition of fibrotic response (Akhmetshina et al., 2012).

Fibroblast growth factor 2 (FGF2), one of the members of the mammalian fibroblast growth factor family, is a cytokine with well-documented cardioprotective effects to attenuate fibroblast activation through antagonizing TGF- β signaling. Svystonyuk et al. (2015) evaluated the effects of low molecular weight fibroblast growth factor (LMW-FGF-2) on myofibroblast-seeded collagen gels, and observed a significant attenuation in TGFβ1 induced myofibroblast activation and ECM remodeling (Fan and Guan, 2016).

Bone morphogenetic protein-7 (BMP7) belongs to the TGF-β super family and is found to have an anti-fibrotic role that is correlated with the inhibition of TGF-β-mediated profibrotic signaling (Hanna and Frangogiannis, 2019). Various studies showed that supplementation with exogenous recombinant BMP-7 can facilitate the reversal of established fibrotic lesions (Tampe and Zeisberg, 2014). Efforts continue to define the therapeutic effectiveness of BMP7 through ongoing *in vitro* and *in vivo* investigations, and to develop small-molecule agonists for systemic administration.

Peroxisome Proliferation Activator Receptors (PPARs) are nuclear transcription factors that form obligate heterodimers with retinoid-X receptors to modulate the transcription of target genes. Preclinical studies have implicated all three PPARs as potential targets for antifibrotic therapy (McVicker and Bennett, 2017).

β-catenin, the central signaling component of the canonical Wnt pathway, is believed to serve a modified target for fibrosis through activation of TGFβ pathway. Hirakawa et al. (2019) evaluated two Wnt/β-catenin signaling inhibitors, ICG-001 and C-82, on the progression of endometriosis. The results suggested that inhibiting the CREB-binding protein (CBP)/β-catenin signal can be a therapeutic target for endometriosis. In another study, Itaba et al. (2019) reported the potent anti-fibrotic therapeutic effect of IC-2, a derivative of ICG-001, on established liver fibrosis.

Recently, several novel components of the TGF β have been discovered, including proprotein convertases (PCSKs), which is in a family of serine proteases that cleave secreted peptides. PCSKs have been reported to be involved in the activation of the TGF-β/Smad signaling pathway. Furin is one of the most ubiquitously expressed PCSK and has been implicated as a potential therapeutic target in fibrosis (Ichiki et al., 2013).

### Rennin–angiotensin–aldosterone system pathway inhibition

The Rennin–angiotensin–aldosterone system (RAAS) plays a critical role in mediating systemic blood pressure, fluid volume, and sodium balance. Overactivity of RAAS is believed to have a pivotal role in the onset of pathological fibrosis. Angiotensin II, the main peptide of the RAS system, has been demonstrated to induce fibrosis in various organs. Many studies reported the critical role of Ang II in the cardiovascular, renal, and pulmonary systems (Murphy et al., 2015) under pathological conditions, such as cardiac hypertrophy and the resultant fibrosis (Murphy et al., 2015). It is now known that angiotensin II acts both independently and in synergy with TGF-β to induce fibrosis via the angiotensin type 1 receptor (AT1) in a multitude of tissues. Ang II can induce fibrosis indirectly by activating proinflammatory mediators such as cytokines, chemokines, adhesion molecules, as well as directly by regulating ECM synthesis and degradation. These studies indicate that therapeutic strategies targeting Ang II are probably an effective way of alleviating fibrosis progression.

The use of RAAS inhibitors, such as renin inhibitors, Angiotensin-converting enzyme (ACE) inhibitors, Angiotensin Receptor Blockers (ARBs), and mineralocorticoid receptor antagonists has been documented to prevent fibrosis and slow the decline of organ function effectively in the process of kidney disease and cardiac hypertrophy (Zhang et al., 2019a). ACE Inhibitors (ACEIs) inhibit the conversion of Ang II from Ang I. A number of ACEIs are currently on the market and used primarily for the treatment of high blood pressure and heart failure. Different ACEIs such as captopril, ramipril, and enalapril, have been extensively tested in both *in vitro* and *in vivo* models and shown effects on reducing fibroblast proliferation, and suppressing collagen deposition and TGF-β1 expression. Similar with ACEIs, ARBs are also frequently prescribed to treat hypertension and have comparable clinical profiles regarding anti-inflammatory and anti-fibrotic outcomes. ARBs compete with the binding of angiotensin II to AT1 receptors, thereby cause angiotensin II receptor blockade. Clinical trials looking at the potential utility of an angiotensin II type 1 receptor blocker (ARB) have demonstrated significant benefit in the prevention of myocardial fibrosis (Zhang et al., 2019a).

Direct Renin Inhibitors (DRIs), Mineralocorticoid Receptor Antagonists (MRAs) as other means of RAAS blockade also have been studied (Zhang et al., 2019a). However, inhibition of RAAS only modestly regresses fibrosis. The combination of different RAAS drugs is an alternative strategy. The use of combination therapy is already established in practice and positive effect has been confirmed.

Developing novel and effective anti-fibrotic therapies through targeting Ang II pathway appears an attractive strategy. Over the past decades, many novel compounds such as kinases inhibitors and hormone peptides showed promising results in alleviating Ang II signaling and compensatory fibrotic remodeling. For example, relaxin, an endogenous peptide hormone in the insulin family, has received much research attention due to the increasing evidence on this peptide hormone’s beneficial effects in fibrotic conditions. Relaxin binds with its endogenous receptor, relaxin family peptide receptor 1 (RXFP1), inhibiting the actions of major pro-fibrotic factors including Ang II (Chow et al., 2014; Samuel et al., 2017).

## Novel interventions

### Biomaterial-based delivery

Current fibrosis therapeutic development technologies are aiming at reducing off-target effects and promoting localized action to affected or fibrotic regions. The development of biomaterial-based delivery methods offers opportunities to improve target specificity and reduce adverse effects. Nano- and micro-particles as carriers for the delivery of can improve transport efficiency and allow more circulation time for the cargo molecules to reach the disease site (Hrkach and Langer, 2020).

Different polymeric microspheres and microparticles have been extensively studied as drug-laden depots to achieve localized delivery of cargos including anti-fibrotic molecules. For example, poly (lactic-co-glycolic acid) (PLGA) microspheres were used to encapsulated resveratrol (RSV) and embedded in a gelatin methacryloyl (GelMA) hydrogel matrix for the treatment of epidural fibrosis (Wang et al., 2021b). Sustained release over weeks was achieved, along with reduced fibroblast proliferation and extracellular matrix deposition. Similarly, low molecular weight heparin (LMWH) loaded PLGA microspheres were administrated in a mouse model of bleomycin-induced lung fibrosis and exerted anti-fibrotic and anti-inflammatory therapeutic effects (Saito et al., 2020). Horejs et al. (2017) developed electrospun polymer matrices incorporated with a fragment of the laminin β1-chain to efficiently modulate MMP expression and activity. They demonstrated that interfacing of the β1-fragment with the mesothelium of the peritoneal membrane *via* a biomaterial abrogates the release of active MMP2 in response to transforming growth factor β1 in a mouse model of peritoneal fibrosis.

In the nanoscale range, there are successful applications of the nanoparticle-based delivery for the treatment of chronic human diseases including cancer, diabetes, and fibrosis. The advances in target-oriented delivery facilitate the therapeutic agents to anchor to their target location for maximized therapeutic function and reduced off-target effects. Controlled systems for delivering therapeutic agents to myofibroblasts have been explored for the treatment of fibrosis (Yazdani et al., 2017). Cell surface ligands such as peptides, antibodies, aptamers, or other moieties are designed against myofibroblast-specific receptors. Activation of hepatic stellate cells (HSCs) is the hallmark of liver fibrogenesis. Targeted delivery of therapeutic agents to activated HSCs has been extensively studied for the treatment of liver fibrosis. Azzam et al. reported a chitosan-nanoparticles (CS-NPs) based delivery system for anti-TGF-β siRNA delivery, promoting delivery into HSCs via modification with PDGF receptor-beta binding peptides. The peptide-modified CS-NPs allow for efficient targeting of fibrotic livers and increased NP uptake by target cells, offering a powerful strategy for improving therapeutic outcomes (Yazdani et al., 2017). In future, targeted delivery to subpopulations of myofibroblasts will remain a highly appealing approach to suppress interstitial fibrosis. However, this field is still in its infancy, as the surface markers delineating these populations are yet to be identified and fully understood.

### Cell therapy

Cell therapy aims to introduce new, healthy cells into a diseased or injured locus, to replace the pathological or missing native cells and to restore organ function. Mesenchymal stromal cells (MSCs) have shown great potential in the treatment of inflammatory diseases and mediating profibrotic factors through their immunoregulatory properties (Usunier et al., 2014). Averyanov et al. (2020) reported the first‐in‐human stem cell therapy in idiopathic pulmonary fibrosis using a high cumulative dose of bone marrow MSCs. The treatment was demonstrated to contribute to an increase in lung function. However, various challenges still exist. Underlying immunocompatibility, stability, heterogeneity, and migratory capacity should be optimized before moving to large-scale clinical trials (Yang et al., 2020).

Chimeric antigen Receptor (CAR) T-cell therapy is a form of immunotherapy that uses engineered T cells to target specific antigens on cancer cells. Patient-derived T cells are collected and modified to produce a modified T cell receptor or CARs on their surface (Aghajanian et al., 2019). Recent advances in CAR-T cell therapy have led to increased interest in their use as a new therapeutic method to address not only cancer but also chronic inflammation associated with fibrosis. Aghajanian et al. (2019) reported the success in using redirected T cell immunotherapy to target pathological cardiac fibrosis in mice. Cardiac fibroblasts that express a xenogeneic antigen were targeted and significantly ablated by the adoptive transfer of antigen-specific CD8^+^ T cells. However, establishing the Car-T cell therapy in humans could take years of development. The therapy is also very expensive and accompanied by severe off-target and side effects. Continued efforts in the identification of antigens that are expressed by activated cardiac fibroblasts may contribute to the development of T cell therapy of fibrosis.

### Gene therapy

Gene therapy has shown promising results in the treatment of a wide spectrum of human diseases. Gene therapy is normally generated by transferring specific genetic information into target cells to correct mutated genes that cause diseases. Many recent technologies, including genetic editing, recombinant DNA technique, mRNA, and miRNA technology, have been utilized in improving the performance of gene therapy in disease context.

MicroRNAs (miRNAs) are a class of small non-coding RNAs that play a role in regulating the expression of messenger RNAs (mRNAs). miRNAs are thought to be involved in diverse biological processes, including fibrosis (Marquez and McCaffrey, 2008). Mediating miRNA by gene therapy provides an attractive approach for therapeutic development of anti-fibrosis drugs. Recent studies have indicated the critical role of a number of microRNAs in the pathogenesis of different forms of human fibrotic disease. For example, Ji et al. (2015) performed genome-wide profiling of miRNA expression in a silica-induced mouse model of pulmonary fibrosis using microarrays. Their results suggested that miR-486-5p is downregulated in the disease model and may act as an anti-fibrotic effector in the development of pulmonary fibrosis.

## Machine learning assisted drug screening

### Machine learning in fibrotic drug discovery and screening

Machine learning (ML) is a rapidly growing field of artificial intelligence research influencing drug screening approaches and enabling new methods of drug discovery (Vamathevan et al., 2019). Generally, ML algorithms are divided into three main categories, supervised, unsupervised, and reinforcement learning. Supervised ML uses labeled datasets to train an algorithm that can later classify data or better predict outcomes of potential inputs to the system (Benjamins et al., 2019). Supervised algorithms include regression analysis, k-nearest neighbour (kNN), Bayesian probabilistic learning, support vector machines (SVMs), random forests and neural networks (Shrestha and Mahmood, 2019). A neural network is one of the most common subsets of machine learning meant to mimic human brain by containing a series of connected nodes, organized in several layers (Yegnanarayana, 2009).Deep learning utilizes neural network approach but contains a much greater quality of hidden layers between data input and output, in comparison to the relatively small number of hidden layers in traditional neural networks (LeCun et al., 2015). Unsupervised ML algorithms analyze and cluster the unlabelled datasets from their potential hidden patterns (Jordan and Mitchell, 2015). Some of the most commonly utilized methods of unsupervised ML are principal component analysis, independent components analysis (ICA), hierarchal learning, data clustering, outlier detection, and singular value decomposition (SVD) (Usama et al., 2019). In reinforcement learning, the algorithm is given a challenge, and is instructed to accomplish a specific goal by optimize a custom function. As the algorithm explores the challenge, it is given a “reward” a representation of our result’s approximate to the desired goal. In this way, the algorithm progressively makes decisions to find the optimal method of reaching the set goal, as it is maximizing the reward (Sugiyama, 2015).

A variety of ML methods were applied to uncover drug candidates or putative targets for drug discovery, for instance the use of support vector machines, optical flow, and tree bagger algorithms, among others. The targets of ML in the drug screening field have focused on analyzing the effects of drugs on tissue contraction, cardiotoxicity, and electrophysiology. Although current drug-centric ML approaches have not specifically focused on anti-fibrosis drugs, we will briefly review several machine learning-based methods for drug discovery and screening applications. We believe these current implementations will help establish the groundwork for future ML studies related to fibrosis as they involve similar assays and datasets.

### Machine learning-based method for drug discovery

In the early stages of drug discovery there are frequently thousands, or tens of thousands of potential candidate molecules. Manual analysis of such tremendous volumes of candidates is extremely labour intensive, thus, recently this process is being enhanced using novel ML methods.

Drug repositioning is a common technique in industry where after research and testing, a molecule is found to be effective on treating a different condition. Due to tremendous volume of approved compounds, ML approaches lend themselves well to determining potential repurposing targets. A novel ML approach for this problem was developed by Wu et al. (2020) with the development of DR AFC (Drug Repositioning based on Anti-Fibrosis Characteristics), which predicts anti-fibrosis drug repositioning candidates through molecular structure and biological profile analysis. Using logistical regression, decision tree, random forest and gradient boosting algorithms, DR AFC was trained on a labelled database of anti-fibrotic and non-anti-fibrotic compounds using molecule structure and biological profile. DR AFC was then applied to experimental drugs in the DrugBank database to locate potential anti-fibrotic drugs for repositioning. DR AFC determined that the molecule Quercetin and the natural compounds of turmeric and curcumin could have potential anti-fibrotic properties based on the activated gene pathways and structure (Wu et al., 2020).

To reduce the large volume of drug candidates, a compound’s molecular structure can be investigated to predict how it may interact with desired biological bind sites. This can be accomplished through computing the standard binding free energy between particular compound and a binding site. Ding and Zhang (2021) developed a platform to determine the standard binding free energy using a deep generative model based on Bennet acceptance ratio, called DeepBAR. Their technology does not require calculation of the intermediate binding steps of the compound, and only requires data regarding the end states of binding sites. The authors tested effectiveness of their system by testing its prediction capabilities of compound binding to the commonly used achiral ring molecule cucurbituril (Sgalla et al., 2018). They found that DeepBAR requires significantly less computational power compared to traditional methods, yet still maintains the high degree of accuracy required for complex testing (Ding and Zhang, 2021).

### Machine learning-based method for assessing tissue contraction

Using the technique of optical flow to measure cardiomyocyte monolayer contractions, Lee et al. (2015) developed a ML-based drug screening platform to identify cardioactive drugs. The ML system was designed to distinguish between the effects of the drugs E-4031, verapamil, and blebbistatin on cardiomyocyte contraction. The motion vector maps of monolayer contraction captured by optical flow were reduced to a single contraction profile using principal component analysis. Twelve parameters were extracted from the contraction profile and used to train a supervised ML algorithm based on the Support Vector Machine (SVM) framework. The trained algorithm categorized the contraction profiles of samples as having either normal or abnormal cardiomyocyte behaviour when exposed to drug treatment. A follow up study by Lee et al. (2017) applied a two-step ML algorithm using SVM to anticipate a cardioactive drug’s mechanism of action on contractility in cardiac tissue strips. In the first step, supervised ML was used to binarily classify each of the 12 compounds as cardioactive or not by observing changes in contractility. The second stage consisted of training a multiclass SVM algorithm with half the compounds to create distinct classification of compounds based on their effects on the cardiac tissue strips. The remaining, unclassified compounds were tested on this algorithm to determine its prediction ability of drug class as an indicator of mechanism of action.

### Machine learning-based method for assessing cardiotoxicity

The effects caused by a compound on the human ether-à-go-go-related gene (hERG) pathway is an important factor when testing for cardiotoxicity as inhibition can cause fatal cardiac arrhythmias (Sanguinetti and Tristani-Firouzi, 2006). To predict if compounds may affect the hERG channel, Cai et al. (2019) developed a multitask deep neural network (DNN) called deephERG. DeephERG operates by using the Molecular Operating Environment (MOE) to generate the properties and vector representation of each drug being analyzed. A subset of this dataset is then input into the multitask DNN for training and validation to determine the cardiotoxicity prediction accuracy. The authors subsequently applied deephERG to analyze 1824 FDA approved drugs for their potential effects on hERG and predicted that 539 candidates could be problematic. DeephERG leverages Mol2Vec descriptors, which is an unsupervised machine learning approach that converts molecular substructures into a vector representation (Cai et al., 2019). Molecules that have comparable substructures end up as similar vectors. Mol2Vec is developed based on application of machine learning on Word2Vec algorithm which a Natural Processing technique for identification and process of closely related molecular substructures and translating them into a vector representation (Jaeger et al., 2018).

A similar study on identifying hERG pathway cardiotoxicity using ML was done by Lee et al. (2019b) through the analysis of molecular physicochemical descriptors associated with cardiotoxicity. After training six different ML algorithms, each was tested on its ability to distinguish cardiotoxicity on a dataset of 2130 compounds. Evaluation of the model showed it had an accuracy of 90.1% at determining potential cardiotoxicity compounds (Lee et al., 2019b).

### Machine learning-based method for electrophysiological assessments

Heylman et al. (2015) created a supervised ML algorithm that compares differences in cardiomyocyte electrical membrane depolarization when treated with propranolol and isoproterenol. A two-photon laser scanning microscope was used to scan individual cardiomyocytes exposed to a fluorescent voltage sensitive dye to extract a membrane depolarization waveform. The TreeBagger random forest algorithm was used as the basis for the supervised ML platform, and functions by comparing the waveform’s upslope, downslope, peak width, maximum height, and plateau height. After training the algorithm to distinguish between treatment groups, the accuracy of group determination for unknown waveforms was found to be 70% for a single depolarization, and 100% for 1-min recordings (Heylman et al., 2015).

A method developed by Juhola et al. (2018) utilized several different supervised ML algorithms such as least-squares-SVM, k-nearest neighbor and random forest to classify calcium transients from patient cardiomyocytes with genetic diseases. The calcium transients were analyzed based on ten different variables extracted from peak information of each sample. These variables include transient peak amplitude, duration, and area under the peak. After training the algorithm, each ML algorithm was applied to a pool of unclassified transients to separate them into the three different diseased groups or as healthy controls. The random forest algorithm was determined to be the most accurate, with 78.6% accuracy at correctly predicting the groups. Despite the fact that the focus of this research was on identification of genetic cardiac diseases, this research may be translatable to investigating changes in calcium transients during drug screening (Juhola et al., 2018).

## Current challenges and future directions in the application of engineered approaches in fibrotic remodeling

The use of OOC disease models, as a viable alternative to traditional pre-clinical testing methods, is improving in verifications with regulatory environment and facing increasing industrial acceptance in the past decade. The upward trend in optimization, regulation, ratification, and adoption of these engineered models promises to lay the foundation of next-generation precise medicine. However, stumbling blocks remain for the widespread adoption of OOC disease models.

### Lineage variability

One of the biggest challenges of hiPSC-based disease modeling is the experimental heterogeneity due to lineage variability and instability. Heterogeneity can result from a multitude of factors such as donor-specific genetic and epigenetic status, as well as clonal variations including incomplete reprogramming and changes during culture. This can hinder reproducibility and confound biological phenotypical differences. In the context of fibrotic disorders, the synergistic interaction of multiple cell types is often involved to manifest specific disease phenotypes. As a result, it is of great importance to construct the model using subpopulations of the same genetic background. The use of standardized differentiation and maturation protocols and cell sorting-based phenotypic selection can potentially minimize variability and thereby increase the reliability of phenotype comparisons in disease modeling. In addition, recent studies describe elegant protocols for the derivation of cardiac fibroblasts from genetically defined hiPSCs (Zhang et al., 2019b). Alternative sources of genetically matched cardiac fibroblasts could be obtained through selective adhesion or cell sorting from the mixed differentiation culture. Other genetically matched cells, such as ECs (Gu, 2018) and macrophages (Sturgeon et al., 2014), can be differentiated from iPSC with established differentiation methods. Isogenic controls, conventionally used in animal models, have been an effective tool for hiPSC-based disease modeling via CRISPR correction of mutated loci. The isogenic cell lines are typically established and cultured in parallel with the fibrosis diseased lines to study epigenetic changes resulting from a specific allele. The disease phenotypes would be corrected in isogenic control and the disease mechanisms can be therefore investigated for potential therapeutic interventions (Merkert et al., 2017). As obtaining hiPSC with disease-causing genetic mutations can be unpredictable and time-consuming, a faster and easier approach is to obtain the healthy iPSC cell lines and CRISPR editing the known mutation into the genome to investigate the epigenetic changes regarding to the specific mutation (Wang et al., 2014; Valley et al., 2019). In comparison to iPSC control from an unaffected family member, the isogenic control would significantly reduce the variability resulting from the different genetic backgrounds and identify the disease relevant molecular changes (Wang et al., 2014; Valley et al., 2019). In future studies, these cells could be used to provide genetically matched cell sources and isogenic controls for robust 3D disease models.

### Tissue maturation

The structural and functional immaturity of hiPSC-derived cells poses another challenge for the direct application of OOC disease models. To this end, enhanced tissue maturation can be achieved using external signals, including electrical, mechanical, and hormonal stimuli. Zhao et al. (2019b) applied electrical stimulation at a gradually increasing intensity to generate electrophysiologically distinct atrial and ventricular tissues with chamber-specific drug responses and gene expression. Ulmer and Eschenhagen (2020) achieved cardiac metabolic maturation through the cultivation of 3D cardiac tissues against a mechanical load, allowing *in vitro* study of metabolic cardiomyopathies. Whilst maturity level in these engineered models is still not on par with the human level, these methods provide a significantly more adult-like hiPSC-derived cardiomyocytes. Continuous effort in improving tissue maturation is essential for developing more physiological relevant disease models.

### Integration of vasculature and local and circulating immune cells

A functional vascular system is a key element in promoting tissue function and evaluating pharmacokinetics and drug effects. However, integrating a vascular interface to multi-OOC systems remains a major challenge without a universal solution. Over the decades, there has been considerable interest in the development of microfluidic OOC platforms with a perfusable vascular network. Co-culture of hiPSC-CMs with endothelial cells can result in primitive vessel-like structures with the potential for *in vivo* anastomosis (Zhang et al., 2018b). The next essential step will be the development of 3D engineered tissue constructs with perfusable vasculature, and include the immune response. Human physiomimetic models that support the cultivation of organ models and circulating immune cells will fundamentally facilitate the study of complex human diseases such as fibrosis, pre-clinical drug development, and precision medicine.

### Scale-up and manufacturing

In terms of platform development, although the culture-based *in vitro* disease models are useful tools to study the disease mechanism and the development of anti-fibrotic drugs, reproducibility, and manufacture of the platform devices are significant challenges. The success of commercial use of organ-on-a-chip model depends heavily on their manufacturability, reproducibility, and availability for large-scale studies. Most lab-used OOC platforms are of relatively low yield due to manual handling in microfabrication and assembly procedures. Standard operation protocol and good manufacture procedures are necessary to develop more standardized and reproducible platform fabrication and end use. Nevertheless, such models are more appropriate to use at a later stage and not suitable for early developmental stage where high-throughput screening for thousands of hits within a limited time window.

The construction of OOC platform involve two key components, the platform fabrication and tissue construction. Platform fabrication relies on microfabrication or microfluidics techniques to build the device for tissue cultivation; tissue construction refers to cultivation of live tissues in a 3D format. The development of more automated and cost-effective manufacturing technologies, such as one-step multi-material 3D printing (134, 135) or hot-embossing (136), can significantly scale up the production of OOC platforms. On the tissue construction side, self-assembly is a widely used process by which cellular components adopt a defined structure without external guidance to achieve complex tissue and organ-level organization and function (75, 137). Additionally, recent advances in 3D bioprinting facilitated multi-scale cell micropatterning and construction of tissues with complex topography (24, 138). These techniques collectively allow rapid and reproducible automated construction of biomimetic organ-like structures. Once the manufacturing process becomes more streamlined and reproducible, widespread adoption of OOC disease models will be accelerated.

## Concluding remarks

While the field is still in the early stage of development, OOC has indeed delivered a breakthrough in disease modeling, allowing for the creation of sophisticated *in vitro* models to understand the processes regulating complex human physiology and pathology. Although the application of these platforms is still confined within the research laboratories, awareness of this technology is spreading fast. The potential for wide adoption across the broader scientific community, industry, and regulatory agencies is encouraging.

Faithfully modelling disease phenotypes and providing clinically meaningful readouts are the important features of an ideal experimental system for drug development. Engineered microtissues, organoids, spheroids, and microfluidic cultures are enjoying rapid growth in the past decade. In terms of fibrosis remodeling, various engineered tools with the capacity to identify pathological factors and potential therapeutics have been devised. Such clinically relevant findings cannot be easily achieved from other commonly used *in vivo* or *in vitro* approaches which are limited by the absence of physiologically relevant hallmarks of human fibrosis.

OOC models are broadly applicable as novel tools for better understating the underlying mechanisms of pathological development of fibrotic diseases, and the inhibition or prevention of fibrotic phenotypes through therapeutic interventions. Each model system exhibits its unique limitations. It may be challenging to find an appropriate model to meet all kinds of experimental requirements and the efficacy of each model is mostly based on the desired assay or translational applications. Current OOC are used to identify the problem and design the system based on the elements needed by this problem, therefore, we do not need everything in the system, but only elements that are critical to reproduce the desired function. The value that OOCs bring is the ability to recapitulate these essential factors *in vitro*. A further boost to these platforms could be achieved using multiple techniques in conjunction, such as hiPSC-derived 3D microtissues on a chip where the real-time properties can be monitored using a buit-in sensor. In addition, automation capacity is strongly desired for chronological tissue assessment and for retrieving useful data from the system. Machine learning applications through analysis of tissue contractions, electrophysiology, calcium flux, and overall toxicity are applicable to fibrosis-related drug screening. In the future, these algorithms could be integrated into anti-fibrosis drug screening pipelines to enhance screening throughput and detection of novel compounds. With highly controlled and formulated micro-environments, these avatars of the human organs can faithfully replicate disease phenotypes. These biosystems present a promising tool with unique features that can propel them to transform the present disease study and drug discovery with superior biofidelity, outperforming the conventional animal and monolayer disease models. Although tumbling blocks still exist in the widespread application of OOC models, we are optimistic that the coherent efforts from the engineers, biologists, and clinicians will entail this complex issue.
